# Exploring the causal relationship between plasma proteins and postherpetic neuralgia: a Mendelian randomization study

**DOI:** 10.3389/fneur.2025.1575941

**Published:** 2025-10-09

**Authors:** Qiuyu Wei, Shaoyong Yu, Yi Luo, Xinghui Song, Pin Qin, Rongji Li, Weichao Sun, Jin Wang, Gang Wu

**Affiliations:** 1 Liuzhou Traditional Chinese Medicine Hospital/Guangxi University of Chinese Medicine, Liuzhou, China; 2 Trauma Joint II Ward, Liuzhou Traditional Chinese Medicine Hospital, Liuzhou, China; 3 Neurosurgery Department, Liuzhou Traditional Chinese Medicine Hospital, Liuzhou, China; 4 Department of Rheumatology, Liuzhou Workers Hospital, Liuzhou, China; 5 Department of Medicine, Guangxi University of Science and Technology, Liuzhou, China; 6 Guangxi University of Chinese Medicine, Nanning, China; 7 Institute office, Liuzhou Traditional Chinese Medicine Hospital, Liuzhou, China

**Keywords:** plasma protein, neuropathic pain, postherpetic neuralgia, Mendelian randomization, drug targets

## Abstract

**Background:**

The proteome represents a critical reservoir of potential therapeutic targets for neurological diseases. This study aims to investigate the causal relationship between plasma proteins and postherpetic neuralgia.

**Methods:**

We performed a two-sample Mendelian Randomization (MR) analysis utilizing genome-wide association study (GWAS) summary statistics from the Decode Genetics dataset (4,907 plasma proteins) and the FinnGen database (490 PHN cases and 435,371 controls). Instrumental variables (IVs) were carefully selected based on stringent criteria to ensure their relevance, independence, and exclusivity. Multiple MR methods, including inverse variance weighting (IVW), MR-Egger, Simple mode, Weighted mode and weighted median, were employed to assess causal relationships. Sensitivity analyses, including leave-one-out analysis, were conducted to confirm the robustness of the findings.

**Results:**

Our analysis identified 14 plasma proteins with significant causal associations with PHN, all *p* < 0.05. Elevated levels of four proteins (NCF1, ATRN, PIANP, and CD48) were associated with an increased risk of PHN, while higher levels of 10 proteins (GABARAPL2, MAP1LC3B, ARF3, KIR2DL5A, DLK1, COLEC12, GPI, SEMG2, EIF4B,and HFE2) were linked to a decreased risk. These findings were supported by sensitivity analyses, which confirmed the robustness of the results and ruled out genetic pleiotropy as a potential bias.

**Conclusion:**

This MR study provides strong evidence for the causal role of specific plasma proteins in the development of PHN. These proteins could serve as potential biomarkers and therapeutic targets for PHN. Future research, including randomized controlled trials, is essential to validate these findings and further explore their clinical applicability.

## 1 Introduction

Postherpetic neuralgia (PHN), a refractory neuropathic pain syndrome resulting from the reactivation of the varicella-zoster virus (VZV), manifests clinically as multidimensional nociceptive sensations including burning, stabbing, lancinating, or paroxysmal electric shock-like pain (1). By definition, PHN persists for ≥3 months following herpes zoster rash resolution, representing the most prevalent chronic sequela of VZV reactivation (2). Epidemiological analyses reveal substantial heterogeneity in reported incidence rates (13–75%), with advanced age and immunosuppression identified as critical risk determinants (3). Notably, this persistent pain syndrome induces marked reductions in quality of life through multifaceted mechanisms: neurophysiological studies demonstrate associations with sleep architecture disruption, mood dysregulation (particularly anxiety and depression), and progressive functional impairment (4). Of clinical significance, severe cases may culminate in complete loss of independent living capacity due to pain-related disability, with documented correlations to suicidal ideation in extreme presentations (5). Given its dual impact on individual health and socioeconomic systems—characterized by prolonged pharmacotherapy, frequent hospitalizations, and productivity losses—elucidating the pathophysiological underpinnings of PHN remains a critical research priority.

Plasma proteins encompass a diverse group of proteins present in the plasma, which fulfill essential physiological functions, including the maintenance of osmotic pressure, facilitation of substance transport, support of immune responses, and provision of nutritional benefits. However, these proteins may also play a significant role in the onset and progression of PHN due to their involvement in neural repair and inflammatory responses (6). In addition, plasma proteins are critical in regulating various biological processes, including molecular pathways, and serve as important drug targets. Recent genome-wide association studies (GWAS) have identified thousands of protein quantitative trait loci (pQTL) associated with plasma proteins (7). These studies have explored the causal effects of plasma proteins on peripheral neuropathic pain, holding promise for identifying potential biomarkers and assessing risk and protective factors related to peripheral neuropathy. A recent randomized controlled trial investigated the relationship between the serotonin transporter gene (5-HTLPR) and the sensitivity and pain severity of trigeminal neuralgia (TN), confirming that 5-HTLPR is associated with both TN sensitivity and the severity of pain (8). Additionally, Kelin et al. examined the correlation between circulating C1Q/tumor necrosis factor-related protein 3 (Ctrp3) concentrations and various metabolic parameters in patients with diabetic peripheral neuropathy (9). Their study revealed that elevated Ctrp3 levels were negatively correlated with nerve conduction velocity, suggesting that Ctrp3 could serve as a predictive marker for peripheral nerve conduction abnormalities. Furthermore, other studies have employed two-sample MR and mediation analysis to establish causal associations between plasma proteins and carpal tunnel syndrome (10). More comprehensively, a study incorporating MR analysis, colocalization analysis, enrichment analysis, protein–protein interaction (PPI) network analysis, and druggable target exploration confirmed the causal relationship between gene-predicted peripheral neuropathy risk and plasma protein abundance, reinforcing the potential of plasma proteins as biomarkers and drug targets for PHN (11). Similarly, given that PHN is a form of peripheral neuropathic pain, it is plausible that it may be closely associated with plasma proteins. Therefore, plasma proteins hold considerable potential for advancing the diagnosis and treatment of PHN.

The pathogenesis of PHN exhibits intricate multifactorial mechanisms, and the etiological interplay between plasma proteomic profiles and PHN development remains incompletely elucidated. A systematic delineation of these causal relationships could substantially advance mechanistic insights into PHN pathophysiology while enabling precision medicine approaches through protein biomarker-guided therapeutic strategies. The synthesis of MR analyses with GWAS and pQTL data establishes a robust framework for early-phase therapeutic target identification. This integrative methodology not only mitigates observational biases through genetic instrumental variables but also enhances causal inference by controlling for confounding factors such as population stratification and environmental covariates. Consequently, this approach optimizes resource allocation, reduces developmental timelines, and expedites the translation of therapeutic discoveries into clinical applications. The exponential growth of plasma proteomic datasets and large-scale GWAS initiatives in PHN research has generated an expanding evidence base for hypothesis validation. Nevertheless, critical knowledge gaps persist regarding the directionality and biological plausibility of protein-disease associations. Therefore, rigorous interrogation of causal plasma protein-PHN relationships through MR as an imperative research priority to disentangle pleiotropic effects and identify clinically actionable targets.

MR is a statistical method based on GWAS data and Mendel’s law of free association to determine the causal relationship between exposure and disease, which can effectively avoid potential environmental confounding by exploiting the random assignment nature of genetic variants (12). This study will use the MR method and utilize genomic data from the Icelandic population to investigate the causal associations between 4,907 plasma proteins and PHN.

## 2 Materials and methods

### 2.1 Study design

**Figure 1:**
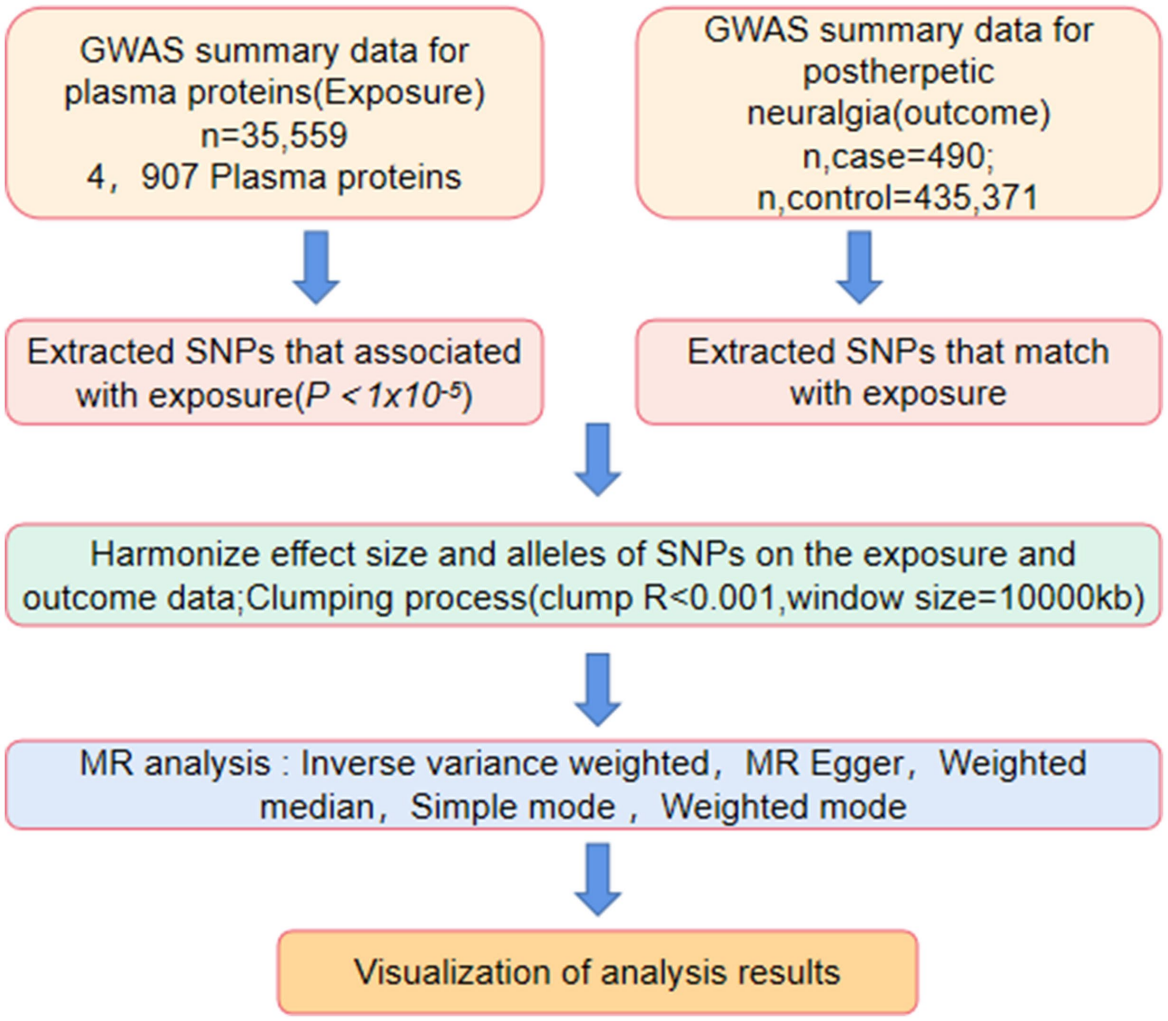
Instrumental variables selection and analysis process flow chart. MR, Mendelian randomization.

The analysis in this study utilized publicly available summary statistics from GWAS, which did not require ethical approval. Figure 1 illustrates the research process. Using the dataset of quantitative trait loci (QTLs) for plasma proteins and summary statistics from large-scale GWAS on PHN, we systematically identified plasma proteins associated with PHN through both GWAS and MR analysis. These steps were taken to further identify potential drug targets for the treatment of PHN.

### 2.2 Data source

#### 2.2.1 Genome-wide association studies summary statistics of plasma proteins

The GWAS summary statistics pertaining to the plasma proteome originate from Decode Genetics,^1^ encompassing a cohort of 35,559 individuals from Iceland (13). Plasma samples from all participants were analyzed utilizing the Soma Scan version 4 assay (SomaLogic), thereby yielding measurements for 4,907 distinct plasma protein levels.

#### 2.2.2 Genome-wide association studies summary statistics of PHN

The GWAS summary statistics for PHN were sourced from the FinnGen database.^2^ The data include 490 cases and 435,371 controls.

#### 2.2.3 Instrumental variables selection

The selected instrumental variables (IVs) should satisfy the three assumptions of MR analysis to ensure its robustness and reliability. Specifically, (1) Relevance: The single nucleotide polymorphisms (SNPs) associated with exposure factors should reach genome-wide significance level (*P_GWAS_* ≤ 1 × 10^−5^). Additionally, to ensure a strong association between the IVs and exposure factors, we calculated the F-statistic for the instrumental variables. Any SNP with an *F*-value below 10 was considered a weak instrument variable prone to bias and was excluded from the analysis. The F-value can be calculated using the formula F = β2/se2 or F = (N-k-1)/k × R2/(1-R2) (14–16). (2) Independence: Subsequently, linkage disequilibrium (LD) among SNPs was removed, as strong LD can lead to bias. SNPs for each plasma protein were clustered, and only independent SNPs were retained. The LD clustering threshold was set to r^2^ = 0.001, with a clustering window size of 10,000 kb. (3) Exclusion restriction: We employed MR-Egger regression, weighted median method, and weighted mode method to eliminate issues of pleiotropy in instrumental variables, ensuring the accuracy of causal inference.

### 2.3 Statistical analysis

We further conducted a two-sample MR analysis and performed a series of other analyses to assess the potential causal relationship of proteins with PHN. The MR analysis follows the STROBE-MR statement, primarily involving the selection of IVs, evaluation of IVs, MR analysis, and sensitivity analysis (17). In the MR analysis, we adopted the inverse-variance weighting (IVW) method. It is worth noting that the random effects model can account for heterogeneity among instrumental variables by allowing for overdispersion in the regression model (18). To further strengthen the validity of the MR results, we conducted MR-Egger, weighted median, simple mode, and weighted mode MR analyses. In brief, MR-Egger can detect and correct for horizontal pleiotropy, where the intercept can be used to identify the presence of horizontal pleiotropy (19). The weighted median method provides consistent estimates even when up to 50% of the information comes from invalid instrumental variables, and it allows for some degree of heterogeneity among the IVs (20). The Simple Mode Method identifies a strongly associated SNP as the instrumental variable, utilizing the effect estimate of this SNP to infer the causal relationship between the exposure and the outcome (21). The Weighted Mode Method addresses differences in genotype frequencies by applying weights to various genotypes, thereby mitigating their influence on the analytical results and enhancing the robustness and reliability of the study findings (22). To assess the heterogeneity of individual causal effects, the Cochran’s Q test was employed (23). Furthermore, the MR-Egger intercept term was utilized to evaluate the presence of horizontal pleiotropy. When the *p*-values from these tests are below the threshold of 0.05, it typically signifies the existence of heterogeneity or pleiotropy. The final MR results were derived from a combination of MR-Egger results and weighted median estimates. In conducting multiple comparisons, we selected IVs with p-values less than 0.05, as determined by three methods—Inverse Variance Weighted, MR-Egger, and Weighted Median—deeming them as significant IVs. This methodology ensured the robustness and reliability of the validation results, thereby enhancing the credibility of the findings. Additionally, we employed R software (version 4.3.2) along with packages such as TwoSampleMR and MendelianRandomization to perform the MR analysis.

### 2.4 Sensitivity analysis

For the sensitivity analysis, we initially fitted the MR-Egger model, subsequently performing additional sensitivity assessments through MR-Egger regression analysis and leave-one-out analysis, with a significant intercept term (*p* < 0.05) considered indicative of horizontal pleiotropy. Consequently, we computed Cochran’s Q statistic to evaluate the heterogeneity among proteins associated with multiple IVs. Finally, a leave-one-out analysis was conducted by re-estimating the MR association after the sequential removal of each variant.

## 3 Results

**Figure 2:**
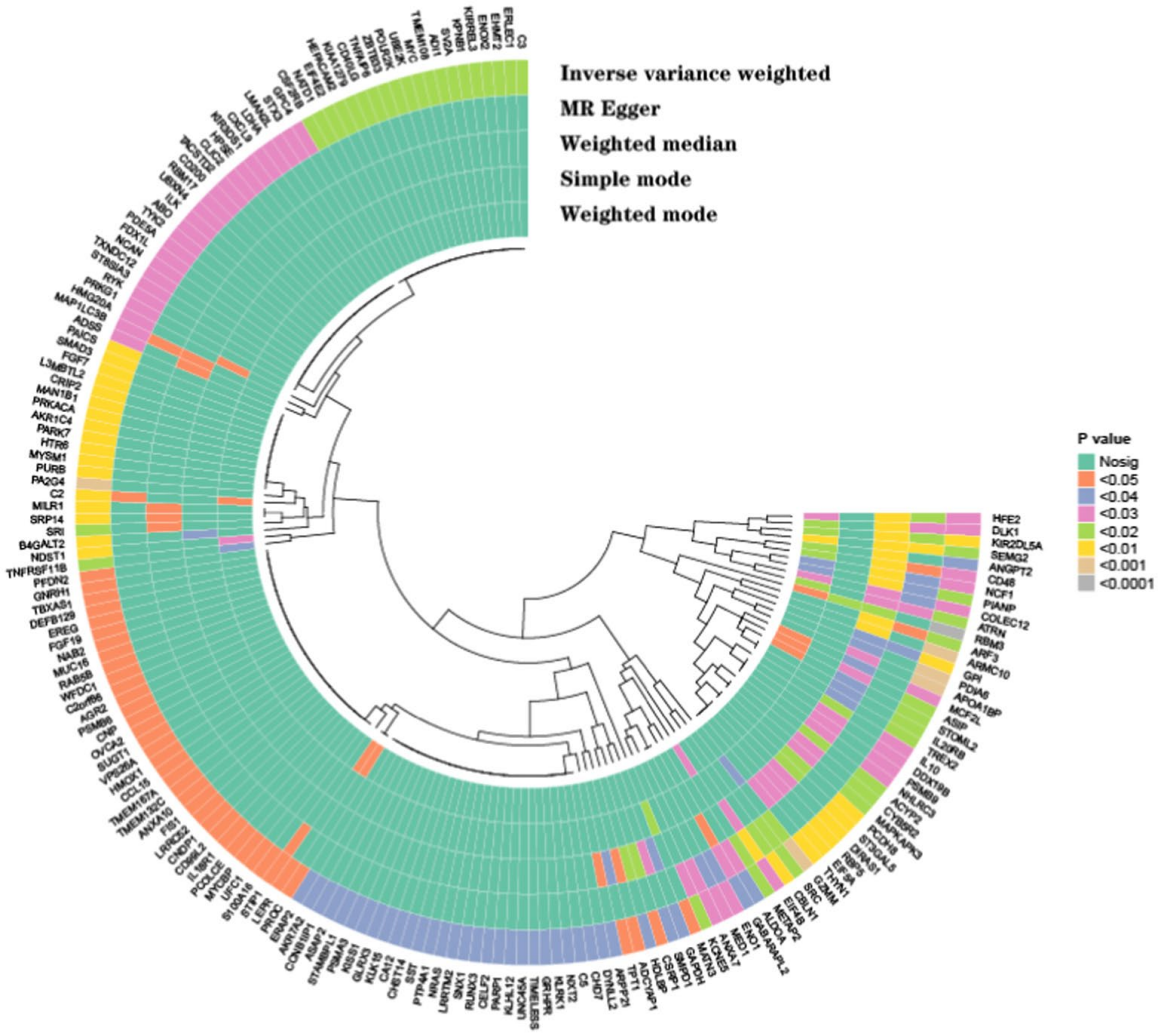
The heatmap displays the causal effect estimates from five MR methods, including IVW, MR-Egger, Weighted Median, Simple Mode, and Weighted Mode, for various genetic variants (SNPs). The *p*-values for each method are color-coded, with a color gradient indicating the statistical significance of the results. The following color scheme was used: no significance (Nosig): represented in green, indicating *p*-values greater than 0.05, *p* < 0.05; represented in yellow *p* < 0.04; represented in light orange *p* < 0.03; represented in dark orange *p* < 0.02; represented in light pink *p* < 0.01; represented in purple *p* < 0.001; represented in dark purple, highlighting highly significant results. Each row represents a genetic variant (SNP), while each column represents a different MR method. The clustering of SNPs based on their similarity in causal effect estimates across methods is visualized via hierarchical clustering, indicated by the dendrogram at the top of the heatmap. This clustering provides an overview of how each SNP’s estimated causal effect compares across different MR methods.

**Figure 3:**
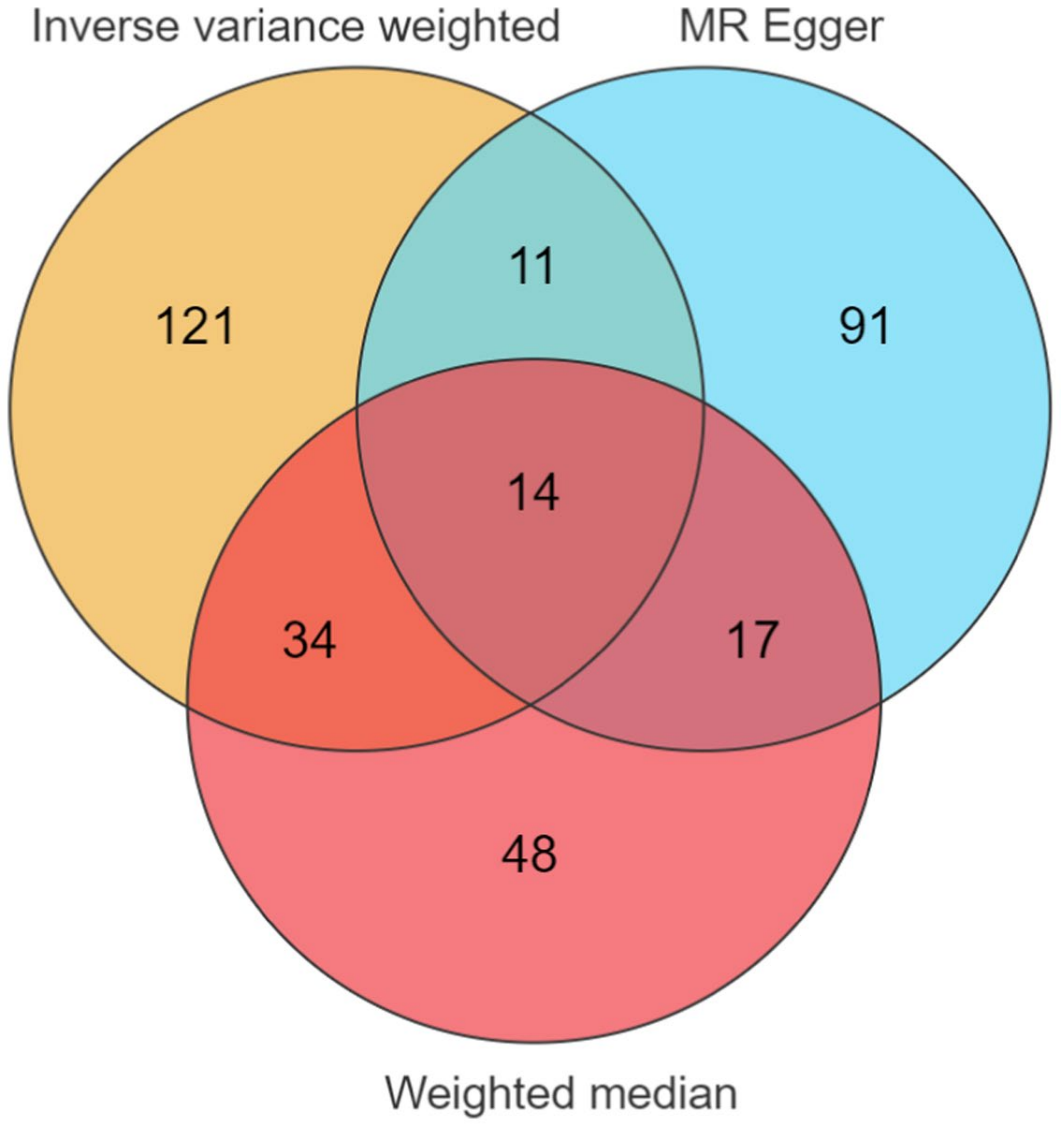
Three different colors represent different methods: orange represents Inverse variance weighted, blue represents MR Egger, red represents weighted median. The number of positive intersection of the three methods is the number of positive plasma proteins. Thus, 14 potential drug target plasma proteins of postherpetic neuralgia can be identified.

**Figure 4:**
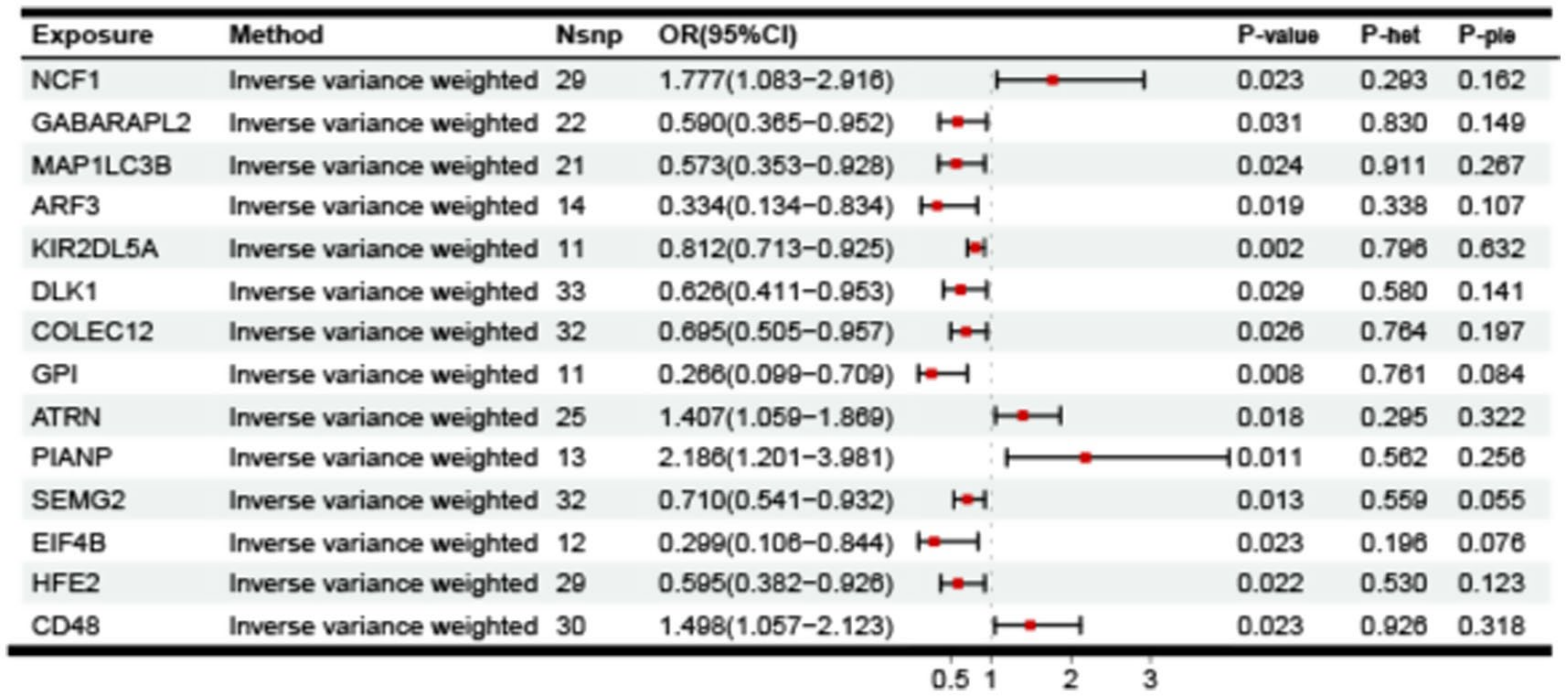
The causal relationship between plasma proteins and PHN in discovery samples. IVW, inverse variance weighted, nSNP, number of instrumental variables, OR, CI, confidence interval.

In this study, we performed MR analysis using two samples to explore the causal relationships between plasma proteins and PHN. We identified 14 significant proteins. The IV F-statistics for all identified proteins were greater than 10, indicating a minimal likelihood of weak IV bias. Based on the results of the forward analysis, we generated a correlation heatmap, a forest plot and venn to visualize our findings (Figures 2–4). These visualizations demonstrate the significant MR analysis results observed in the discovery sample.

As shown in Figure 2, elevated levels of 4 proteins: NCF1 (*p* = 0.023, 95%CI = 1.777 [1.083–2.916]), ATRN (*p* = 0.018, 95CI = 1.407 [1.059–1.869]), PIANP (*p* = 0.011, 95%2.186 [1.201–3.981]), CD48 (*p* = 0.023, 95%CI = 1.498 [1.057–2.123]), were associated with an increased risk of PHN (OR range from 1.407 to 2.186). In contrast, elevated levels of 10 proteins: GABARAPL2 (*p* = 0.031, 95% CI = 0.590 [0.365–0.952]), MAP1LC3B (*p* = 0.024, 95% CI = 0.573 [0.353–0.928]), ARF3(*p* = 0.019, 95% CI = 0.334 [0.134–0.834]), KIR2DL5A (*p* = 0.002, 95% CI = 0.812 [0.713–0.925]), DLK1 (*p* = 0.029, 95% CI = 0.626 [0.411–0.953]), COLEC12 (*p* = 0.026, 95% CI = 0.695 [0.505–0.957]), GPI (*p* = 0.008, 95% CI = 0.266 [0.099–0.709]), SEMG2 (*p* = 0.013, 95% CI = 0.710 [0.541–0.932]), EIF4B (*p* = 0.023, 95% CI = 0.299 [0.106–0.844]), HFE2 (*p* = 0.022, 95% CI = 0.595 [0.382–0.926]), were associated with a decreased risk of PHN (OR range from 0.266 to 0.812).

**Figure 5:**
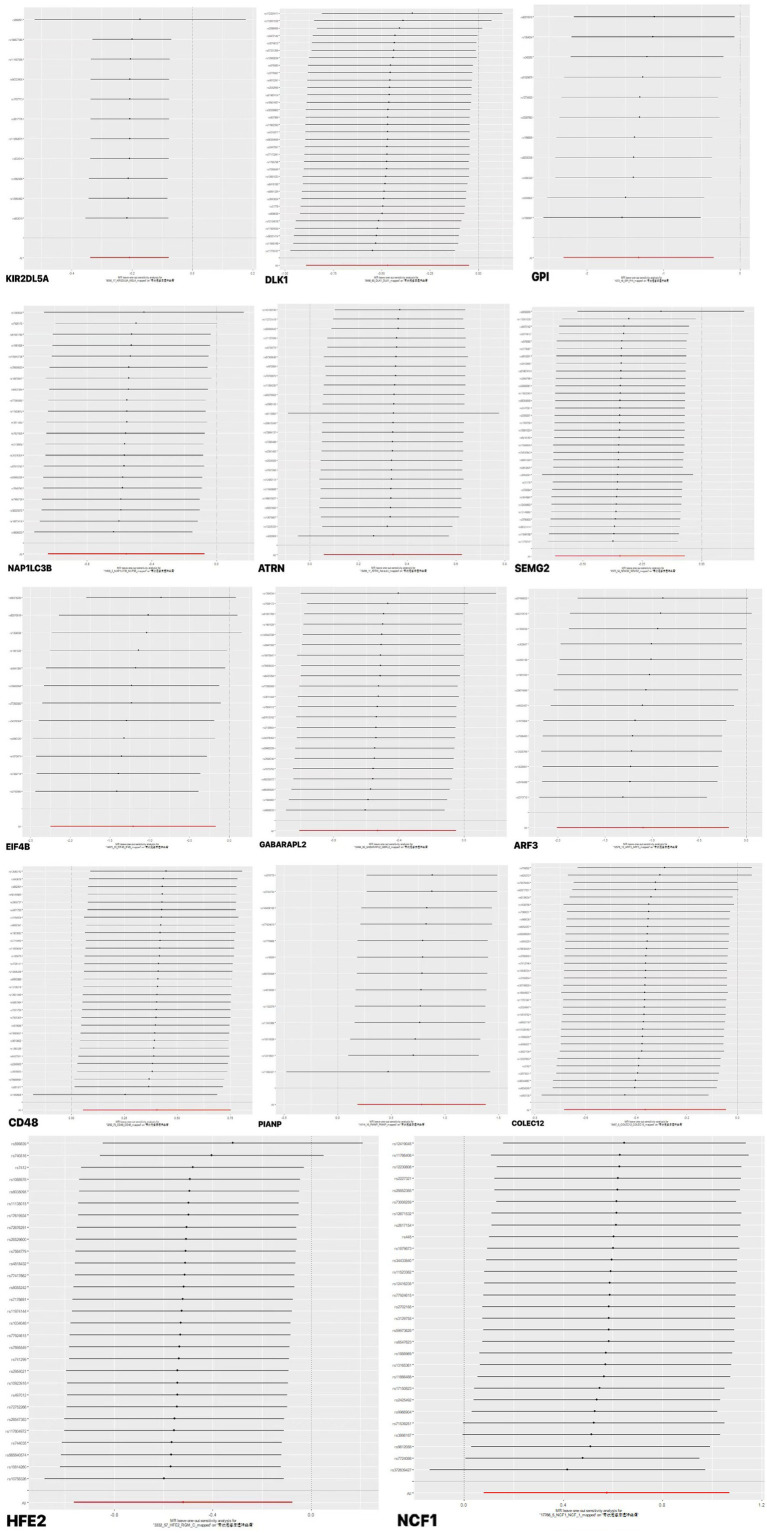
Forward MR leave-one-out plot for the causal association between 14 plasma proteins and PHN. The vertical axis shows the SNPs included in the analysis.

In sensitivity analyses, the results of leave-one-out analyses proved that the positive MR was reliable. Leave-one-out sensitivity analyses were performed for each of the SNPs to determine the causal effect of the 14 plasma proteins on PHN. We found that the results were on the side of the zero line regardless of the exclusion of any of the SNPs (Figure 5). Genetic pleiotropy did not bias the results according to the MR-Egger regression intercept method. However, no causal relationships were found between the remaining 4,893 plasma proteins and PHN.

## 4 Discussion

In our study, MR analysis was employed to examine the associations between plasma proteins and the risk of PHN. The primary objective was to explore the genetic evidence of a potential causal relationship between plasma proteins and the risk of PHN. Through MR analysis, we identified 14 plasma proteins (NCF1, ATRN, PIANP, CD48, GABARAPL2, MAP1LC3B, ARF3, KIR2DL5A, DLK1, COLEC12, GPI, SEMG2, EIF4B, and HFE2) with causal relationships to PHN. In contrast, there was no significant correlation between PHN and the remaining plasma proteins. These findings contribute valuable insights into the pathophysiology of PHN and highlight potential biomarkers for early diagnosis and therapeutic targets.

PHN may persist for extended periods, ranging from several months to even years, significantly diminishing the quality of life for affected individuals (24). In addition to this, PHN often causes profound disruptions across various domains, including physiological, psychological, and social aspects. The underlying pathogenesis of PHN is multifaceted, involving several interconnected biological processes such as nerve injury and regeneration, inflammatory responses, central sensitization, and genetic predisposition (4, 25, 26). Despite this complexity, the early diagnosis of PHN remains challenging and is often insufficient, relying on clinical evaluation, imaging tests, and neurophysiological assessments (27). While clinical evaluation is the most frequently employed diagnostic approach, it is inherently dependent on the physician’s expertise and the patient’s subjective reporting, which can lead to diagnostic inaccuracies. Moreover, in the early stages of PHN, imaging changes are frequently subtle or absent, making detection difficult. Neurophysiological tests, although informative, are often constrained by the need for specialized equipment and trained personnel, limiting their practical application in routine clinical settings. Consequently, early diagnostic methods still lack the necessary specificity and sensitivity, leading to a higher likelihood of misdiagnosis or delayed diagnosis, particularly during the early phase of PHN.

At present, treatment strategies for PHN primarily involve pharmacological and interventional methods (28). Pharmacological therapies commonly used include tricyclic antidepressants (such as nortriptyline and amitriptyline), anticonvulsants (such as pregabalin and gabapentin), opioid analgesics (including oxycodone, morphine, and fentanyl), topical analgesics (e.g., lidocaine patches), and neurotrophic agents (such as methylcobalamin and vitamin B1) (29–34). These medications act on pain pathways through various mechanisms, with the goal of effectively reducing pain and enhancing the patient’s overall quality of life. Consequently, the choice of an appropriate therapeutic regimen is guided by the patient’s response to these different treatment options. In terms of interventional treatments, a variety of techniques are commonly employed in clinical practice, including nerve blocks, ozone injections, radiofrequency thermal coagulation, and pulsed radiofrequency (35). These interventions directly target neural structures, effectively alleviating pain and improving the patient’s functional status (33, 36, 37). In addition to pharmacological and interventional approaches, several adjunctive therapies, such as physical therapy, psychotherapy, and traditional Chinese medicine, are widely used in the management of PHN (38, 39). These complementary treatments not only assist in pain relief and the improvement of psychological well-being but also enhance the overall therapeutic outcome. However, despite the availability of diverse treatment options, the overall efficacy remains limited, with potential risks of dependence, addiction, and other adverse effects (40, 41). Therefore, further research into the molecular mechanisms of PHN and the identification of new therapeutic targets is crucial in order to optimize pain management and improve patients’ quality of life.

To address the existing research gap, several studies have explored the relationship between PHN and plasma proteins, uncovering potential biomarkers and therapeutic targets. Research has indicated that certain inflammatory markers, such as C-reactive protein and albumin, can serve as early diagnostic indicators for predicting the onset of PHN, particularly in patients with acute herpes zoster (42). Additionally, the application of mass spectrometry has revealed differentially expressed proteins in plasma, which could provide important clues for the early diagnosis of PHN and help identify key molecules associated with nerve injury and chronic inflammation (43). Furthermore, recent studies have examined the potential use of platelet-rich plasma (PRP) in the treatment of PHN, suggesting that PRP may improve clinical symptoms by promoting nerve regeneration and alleviating pain (44). Transcriptomic analyses of cerebrospinal fluid and blood have shown significant immune responses and neuropathological changes in PHN patients, with prominent alterations in cytokine and transcriptomic marker expression (45). Therefore, inflammatory markers, differentially expressed proteins, and the application of PRP offer new perspectives for the early diagnosis and treatment of PHN, holding promise for advancing both research and clinical practices in this field.

MR analysis can be used to explore the causal relationship between plasma proteins and PHN. Compared to other analytical methods, this study reduces the impact of confounding bias by utilizing genetic variation, which is randomly assigned during fertilization and independent of environmental factors. The genetic variations in 4,907 plasma proteins were obtained from the most recent GWAS data, ensuring the stability and accuracy of the instrumental variables in the MR analysis. Moreover, the exposure and outcome data used in this study were derived from non-overlapping public datasets, effectively minimizing potential bias during the experimental process. By using genes as instrumental variables, MR analysis provides a clearer understanding of the causal relationship between exposure factors and outcomes while reducing the likelihood of reverse causality.

However, this study has several limitations. First, the data used in this study were derived exclusively from individuals of European ancestry, which may limit the generalizability of the results due to potential genetic heterogeneity across different populations. Second, although random allocation of genetic variation helps reduce some confounding, unmeasured confounding factors may still influence causal inferences. Finally, the sample size was not sufficiently large, which led to the use of instrument variables that did not meet the traditional GARS significance threshold (*p < 1 × 10^−06^*). Hence, further studies with larger sample sizes and more diverse populations are required to validate these findings.

## 5 Conclusion

This MR study suggests that genetic variations in NCF1, ATRN, PIANP, CD48, GABARAPL2, MAP1LC3B, ARF3, KIR2DL5A, DLK1, COLEC12, GPI, SEMG2, and EIF4B are causally associated with postherpetic neuralgia (PHN). These plasma proteins may serve as potential biomarkers, drug targets, and disease indicators for PHN. Consequently, these findings provide valuable insights and new directions for further exploration of the pathogenesis of PHN, its early diagnosis, and targeted therapies. However, it is crucial to conduct further rigorous randomized controlled trials (RCTs) to fully assess the practicality and effectiveness of these plasma proteins and to validate the current results.

## Data Availability

The summary data of plasma protein can be downloaded from the website https://www.decode.com/summarydata/. The summary data of FINNGEN can be downloaded from the website https://www.finngen.fi/en/access.

## Footnotes

^1^ https://www.decode.com/summarydata/

^2^ https://www.finngen.fi/en/access_results
