# Correction: Exploring the causal relationship between plasma proteins and postherpetic neuralgia: a Mendelian randomization study

**DOI:** 10.3389/fneur.2025.1726757

**Published:** 2025-11-10

**Authors:** Qiuyu Wei, Shaoyong Yu, Yi Luo, Xinghui Song, Pin Qin, Rongji Li, Weichao Sun, Jin Wang, Gang Wu

**Affiliations:** 1 Liuzhou Traditional Chinese Medicine Hospital/Guangxi University of Chinese Medicine, Liuzhou, China; 2 Trauma Joint II Ward, Liuzhou Traditional Chinese Medicine Hospital, Liuzhou, China; 3 Neurosurgery Department, Liuzhou Traditional Chinese Medicine Hospital, Liuzhou, China; 4 Department of Rheumatology, Liuzhou Workers Hospital, Liuzhou, China; 5 Department of Medicine, Guangxi University of Science and Technology, Liuzhou, China; 6 Guangxi University of Chinese Medicine, Nanning, China; 7 Institute Office, Liuzhou Traditional Chinese Medicine Hospital, Liuzhou, China

**Keywords:** plasma protein, neuropathic pain, postherpetic neuralgia, Mendelian randomization, drug targets

In the published article, the affiliation “Guangxi University of Chinese Medicine, Nanning, China” was omitted from the paper. This affiliation has now been added to author Weichao Sun.

Author Qiuyu Wei was erroneously assigned as corresponding author. The correct corresponding authors are Gang Wu and Jin Wang.

The **Abstract** in the published article was corrected. The original version appears below: “**Background:** The proteome represents a valuable resource for identifying therapeutic targets and clarifying disease mechanisms in neurological disorders. This study investigated potential causal relationships between plasma proteins and postherpetic neuralgia (PHN).

**Methods:** We conducted a two-sample Mendelian randomization (MR) analysis using genome-wide association study (GWAS) summary statistics from the Decode Genetics dataset (4,907 plasma proteins) and the FinnGen database (490 PHN cases and 435,371 controls). Instrumental variables (IVs) were selected based on relevance, independence, and exclusivity. Causal associations were assessed using inverse-variance weighted (IVW), MR-Egger regression, simple mode, weighted mode, and weighted median methods. Sensitivity analyses, including leave-one-out tests, evaluated result robustness, while colocalization analysis examined shared causal variants between traits.

**Results:** Eight plasma proteins showed significant associations with PHN (PFDR < 0.05). Higher levels of ATRN, PIANP, and CD48 correlated with increased PHN risk, whereas elevated KIR2DL5A, GPI, SEMG2, EIF4B, and HFE2 levels were associated with reduced risk. Sensitivity analyses supported these findings and excluded genetic pleiotropy as a major confounding factor. Colocalization analysis did not detect shared causal variants (PPH4 < 0.8).

**Conclusion:** These results suggest a potential causal role for eight plasma proteins in PHN pathogenesis. While these proteins may serve as biomarkers or therapeutic candidates, further validation is required. This study advances understanding of PHN pathophysiology and supports future investigations into diagnostic and therapeutic strategies.”

The updated version appears below:

“**Background:** The proteome represents a critical reservoir of potential therapeutic targets for neurological diseases. This study aims to investigate the causal relationship between plasma proteins and postherpetic neuralgia.

**Methods:** We performed a two-sample Mendelian Randomization (MR) analysis utilizing genome-wide association study (GWAS) summary statistics from the Decode Genetics dataset (4,907 plasma proteins) and the FinnGen database (490 PHN cases and 435,371 controls). Instrumental variables (IVs) were carefully selected based on stringent criteria to ensure their relevance, independence, and exclusivity. Multiple MR methods, including inverse variance weighting (IVW), MR-Egger, Simple mode, Weighted mode and weighted median, were employed to assess causal relationships. Sensitivity analyses, including leave-one-out analysis, were conducted to confirm the robustness of the findings.

**Results:** Our analysis identified 14 plasma proteins with significant causal associations with PHN, all *p* < 0.05. Elevated levels of four proteins (NCF1, ATRN, PIANP, and CD48) were associated with an increased risk of PHN, while higher levels of 10 proteins (GABARAPL2, MAP1LC3B, ARF3, KIR2DL5A, DLK1, COLEC12, GPI, SEMG2, EIF4B, and HFE2) were linked to a decreased risk. These findings were supported by sensitivity analyses, which confirmed the robustness of the results and ruled out genetic pleiotropy as a potential bias.

**Conclusion:** This MR study provides strong evidence for the causal role of specific plasma proteins in the development of PHN. These proteins could serve as potential biomarkers and therapeutic targets for PHN. Future research, including randomized controlled trials, is essential to validate these findings and further explore their clinical applicability.”

The original version of this article has been updated.

